# Non-Synergistic Effect of *Trichoderma harzianum* and *Glomus* spp. in Reducing Infection of Fusarium Wilt in Banana

**DOI:** 10.3390/pathogens8020043

**Published:** 2019-03-31

**Authors:** Arfe G. Castillo, Cecirly G. Puig, Christian Joseph R. Cumagun

**Affiliations:** 1 Philippine Science High School-Cordillera Administrative Region Campus, Purok 12, Irisan Baguio City 2600, Philippines; agcastillo3@up.edu.ph; 2 Institute of Crop Science (ICropS), College of Agriculture and Food Science, University of the Philippines Los Baños, College, Laguna 4031, Philippines; 3 Institute of Weed Science, Entomology and Plant Pathology, College of Agriculture and Food Science, University of the Philippines Los Baños, College, Laguna 4031, Philippines; cecirly.puig@usep.edu.ph; 4 College of Agriculture and Related Sciences, University of Southeastern Philippines (USeP), Tagum City, Davao del Norte 8100, Philippines; 5 Molecular Phytopathology and Mycotoxin Research, University of Göttingen, Grisebachstrasse, 637077 Göttingen, Germany

**Keywords:** *Glomus* spp., *Trichoderma harzianum*, *Fusarium oxysporum* f. sp. *cubense* Tropical Race 4, ‘Lakatan’ banana, Panama wilt

## Abstract

Philippine banana is currently threatened by *Fusarium oxysporum* f. sp. *cubense* Tropical Race 4 (FocR4). This study investigated the use of *Trichoderma harzianum* pre-treated with *Glomus* spp, as a means of managing Fusarium wilt on young ‘Lakatan’ banana seedlings. Results showed that *Glomus* applied basally significantly improved banana seedling growth with increased increment in plant height and pseudostem diameter and heavier root weight. The application of *Glomus* spp. alone offered 100% protection to the ‘Lakatan’ seedlings against FocR4 as indicated by the absence of the wilting symptom. A combination of *T. harzianum* and *Glomus* spp. also gave significant effect against Fusarium wilt through delayed disease progression in the seedlings but was not synergistic. Competitive effects were suspected when application of the two biological control agents on banana roots was done simultaneously.

## 1. Introduction

Philippine banana, particularly ‘Cavendish’ type, has gained popularity worldwide making the country as the third largest producer and exporter (in terms of volume) in the world in 2012 after India and Brazil [1]. All commercially important cultivars, such as ‘Grand Naine’ and ‘Gros Michel’ and the top-selling local cultivar ‘Lakatan’, succumbed to infection by *Fusarium oxysporum* f. sp. *cubense* Tropical Race 4 (FocR4) which had been reported in other Asian countries. Fusarium wilt, also known as Panama wilt, a soil-borne fungal disease has been considered a threat to the Philippine banana industry, with Tropical Race 4 being the most devastating [2]. A quarantine approach has been implemented to address the rapid spread of the disease. The most affected stakeholders are the independent banana growers since multinationals can contain and manage the disease.

Until today, there is no single measure to control the disease. Cultural and chemical methods have not yielded much success and have proven to be uneconomical, time-consuming, labor-intensive and environmentally hazardous, especially measures involving soil fumigation with chemicals. Hence, the search for an alternative strategy such as biological control is underway. *Trichoderma harzianum* was selected for use as a mycoparasite and can be frequently found in banana rhizosphere microflora [3]. *Trichoderma* species can control diseases caused by various fungal pathogens through their various types of action which include mycoparasitism, antibiosis, induced resistance, competition for nutrients or space, and inactivation of the pathogen’s enzymes [4,5]. Wibowo et al. [6] used *T. harzianum* to control Fusarium wilt of banana both in vitro and in vivo. The in vitro test was promising but the in vivo test resulted in an insignificant reduction in disease intensity induced by *F. oxysporum* f. sp. *cubense*. Along with *T. harzianum*, Zhang et al. [7] used matured compost mixed with antagonists *Paenibacillus polymyxa* and two *Bacillus* species and obtained up to 80% reduction in Fusarium wilt incidence, enhanced plant growth and stimulation of antifungal enzymes. To enhance the biocontrol ability of *T. harzianum*, Thangavelu et al. [8] utilized organic substrates. Of the five different substrates, dried formulation of banana leaves allowed increased propagule of *T. harzianum* which effectively controlled Fusarium wilt comparable to that of the fungicide carbendazim.

In addition, the vesicular arbuscular *Glomus* spp. was tested in this study as it is known to reduce access sites of root-invading pathogens and stimulate host defense and indirectly suppress plant pathogens through enhanced nutrition by the secretion of growth-promoting substances, increased lignifications and production of the antifungal compounds chitinase and isoflavonoids [9,10,11,12]. When used singly, under different sources of nutrients, banana seedlings tolerated Panama wilt but later increased because of mineral fertilization [13]. The combination of the two fungi could have a synergistic effect on the reduction of Fusarium wilt of banana. This study investigated the use of *T. harzianum* as a means of managing Fusarium wilt on young banana plants pre-treated with *Glomus* spp.

## 2. Materials and Methods

### 2.1. Source of FocT4, Fungal Biocontrol Strains Cultures and Banana Seedlings

A pure isolate of FocR4 in Potato Dextrose Agar (PDA) medium was obtained from the culture collection of the Plant Pathology Research Laboratory, University of Southeastern Philippines, Mabini Campus, Compostela Valley Province. Tropical Race 4was previously confirmed from the existing ‘Lakatan’ production area infected by Fusarium wilt. One-month-old tissue culture seedlings of the susceptible ‘Lakatan’ cultivar were purchased from the Institute of Plant Breeding (IPB), University of the Philippines Los Baños (UPLB). The seedlings were transferred in 1:1 garden soil and coco-coir medium pre-inoculated with *T. harzianum* with a concentration of 3 × 10^5^ conidia per gram of the commercial BioQuick. The conidial concentration was measured using a hemacytometer, The set-up used a double pot system in order to contain the FocR4-infected soil. *Glomus* spp. used in this study was a commercial Vesicular Arbuscular Mycorrhiza Root Inoculant or VAMRI, chopped dried corn roots infected with arbuscular mycorrhizal fungus, either *Glomus mosseae* or *Glomus fasciculatum* [14]. A recommended rate of 5 g of the product per plant was put as basal during transplanting. Two weeks later, FocR4 was introduced with the concentration of 1 × 10^5^ per gram of the substrate. Both BioQuick and VAMRI are products of the National Institute of Biotechnology and Molecular Biology (BIOTECH), UPLB. The experiment was conducted in a non-controlled greenhouse during the summer months (March-May) of 2016. Under such condition, plants were watered twice daily (morning and afternoon) and fertilized weekly using NPK (14-14-14) in the first week and urea (46-0-0).

### 2.2. Soil Treatments

To evaluate the effectiveness of the two biocontrol fungi against Fusarium wilt of banana, seven treatments were tested under non-controlled greenhouse at the Institute of Weed Science, Entomology and Plant Pathology, UPLB. The following treatments were applied: T1 = FocR4 alone, T2 = FocR4 + *T. harzianum,* T3 = FocR4 + *Glomus* spp, T4 = FocR4 + *T. harzianum* + *Glomus* spp. T5= *T. harzianum* alone, T6 = *Glomus* spp. alone and T7 = *T. harzianum* and *Glomus* spp. Nine replicates (= number of plants) per treatment were used. 

### 2.3. Data Collection and Statistical Analysis

The effect of the treatments on plant growth and vigour was also estimated after six weeks by measuring plant height, pseudostem diameter and root weight. Height and stem diameter increment/loss were computed as the difference between the final and initial data taken during the experiment. The effectiveness of the artificially induced suppressive soil was assessed based on disease incidence (%) and disease progression, estimated by using the area under the disease progress curve (AUDPC) which was compared statistically among treatments [15] every week on a six-week period. Sample plants were sectioned longitudinally to confirm the presence of vascular lesions characteristic of FocR4 infection. The experiment was repeated once and the experimental design followed a completely randomized design. Data collected were analyzed using the Analysis of Variance and significant mean differences were separated using Tukey’s Honest Significant Test (HSD) in SPSS 14.0.

## 3. Results and Discussion

### 3.1. Reaction of ‘Lakatan’ Banana Seedlings to Biocontrol Application

Growth parameters of ‘Lakatan’ seedlings as affected by the application of *T. harzianum* and *Glomus* spp. were compared (Table 1). Data showed that *Glomus* spp. and *T. harzianum* applied alone significantly increased plant height and pseudostem diameter and gave heavier root weight than the untreated control. 

In FocR4 inoculated plants, *Glomus* spp. applied alone showed a significant positive effect on seedling growth as indicated by a comparable effect with the non-inoculated plants. This indicates protection of *Glomus* spp. from FocR4 infection resulting in healthy seedlings. A different result, however, was observed when *Glomus* spp. was combined with *T. harzianum* and its sole application. Seedling growth was significantly lower. This can be explained by the infection of FocR4 in the sampled plants which affected plant growth.

According to Smith and Read [16], the beneficial effect of mycorrhizal associations is the enhanced uptake of mineral nutrients. Mycorrhizal symbiosis is frequently associated with increased photosynthetic rates of mycorrhizal plants. Although the increased nutrient uptake is the most significant benefit of mycorrhizae, this fascinating symbiotic relationship offers the following benefits to the host plants: (1) enhances plant efficiency in absorbing water, (2) reduces fertilizer requirement, (3) increases drought resistance (4) increases pathogen resistance, (5) protects against damage from heavy metals and other pollutants; improves seedling growth and survival; minimizes various plant stresses, (6) improves soil structure and contributes to nutrient cycling processes and (7) contributes toward carbon sequestration. The abilities of vesicular arbuscular mycorrhiza to promote growth opened new perspectives for the use of these fungi especially as root inoculants in banana nurseries.

### 3.2. Effect on Fusarium Wilt Incidence and Disease Progression

**Figure 1:**
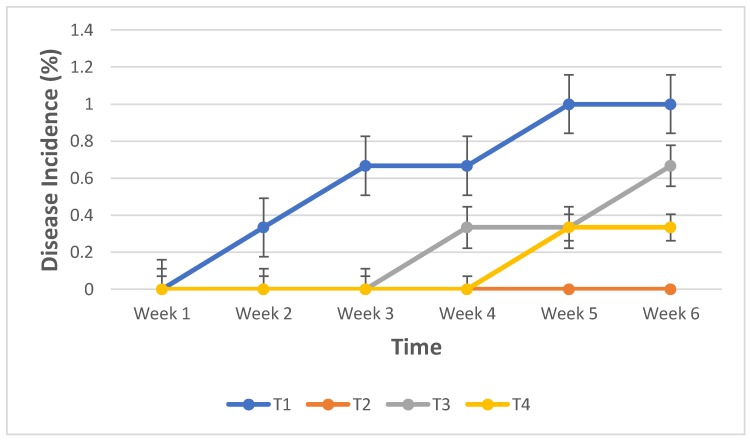
Progression of Fusarium wilt on banana seedlings of cv. ‘Lakatan’ under experimental conditions: T1- FocR4, T2- FocR4 + *T. harzianum*, T3- FocR4 + *Glomus* spp. and T4- FocR4 + *T. harzianum* + *Glomus* spp. Error bars represent standard error of the mean.

**Figure 2:**
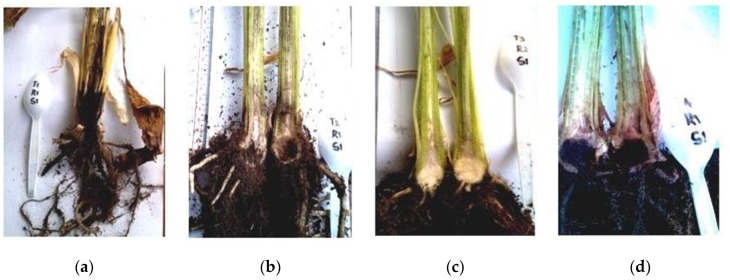
Longitudinal sections of the FocR4-inoculated sample plants showing characteristic lesions caused by infection of the fungus on the vascular tissues: (**a**): untreated; (**b**): *T. harzianum*; (**c**): *Glomus* spp.; and (**d**): combination of *T. harzianum* and *Glomus* spp.

At six weeks post inoculation, Fusarium wilt incidence varied with the treatments. The application of *Glomus* spp. alone offered 100% protection to the ‘Lakatan’ seedlings against FocR4 as indicated by the absence of the wilting symptom (Figure 1). Combination of *T. harzianum* and *Glomus* spp. also gave significant effect towards Fusarium wilt through delayed disease progression in the seedlings compared to the untreated control (Table 2). The presence of *Glomus* spp., *T. harzianum* or both had an effect on FocR4, with *Glomus* spp. applied solely being the most efficient treatment (Figure 2).

Disease incidence of seedlings increased over time. Despite the encouraging results, disease suppression by the presence of *T. harzianum* was less effective, probably due to soil conditions like moisture, pH and even saprophytic competition for nutrients and sites with FocR4 and with *Glomus* spp. This was unexpected as combined inoculation of *T. harzianum* and *G. intraradices* resulted in a general synergistic effect on control of Fusarium wilt of melon than for plants inoculated with either biocontrol agent singly [17]. The same authors also found that co-inoculation of plants with the arbuscular mycorrhizal fungus and *T. harzianum* provided more effective control of Fusarium wilt than each arbuscular mycorrhizal fungus inoculated alone, but with an effectiveness similar to that of *T. harzianum* inoculated plants [18,19]. The interaction of the two fungi with the plant induced hormone production such as salicylic acid, jasmonic acid and ethylene, as compared to their treatment singly [20]. In India, the combination of *G. mosseae* and *T. harzianum* when challenged with *Fusarium* under field conditions could provide 61 and 70% increase in banana plant height and girth, respectively, and 75% in bunch weight over untreated plants with the pathogen alone [15]. A single application of *Gigaspora margarita* was found to be effective in protecting banana plantlets against *F. oysporum* f. sp. *cubense* only when applied 60 days, before they were inoculated with the pathogen [21]. This work clearly showed the protective mechanism of mycorrhizae on the banana roots and the importance of root establishment for effective control. 

In contrast, the non-synergistic effect of the combined application of *T. harzianum* and *Glomus* spp. in controlling disease incidence of Fusarium wilt of banana was observed. There was a case similar to our findings that dual inoculation of cacao seedlings with *T. asperellum* and arbuscular mycorrhizal fungi *Gigaspora margarita* and *Acaulospora tuberculata* was not effective in controlling black pod disease caused by *Phytophthora megakarya* [22]. We surmised that in the combined treatment of the two biocontrol agents, pre-inoculation of the soil with *T. harzianum* interfered with the effects of *Glomus* spp., although *Glomus* spp was applied two weeks before with the pathogen. The treatment of *Glomus* spp. two weeks before the pathogen was not enough for its establishment in the banana roots and four weeks after transplanting, the disease incidence had increased, which could be due to the competitive effects of *T. harzianum.* It was clear that there was hardly a disease symptom on the banana roots when treated alone with *Glomus* spp. According to Gilbert and Parker [23], the co-existence of two closely related microbial species could assist in selecting more effective biological control agents. Although *Glomus* and *Trichoderma* share more or less same physiology, combined treatment of the two fungi was not recommended as shown by our results but contrary to other studies [17,21,24]. Recently, Manasfi et al. [25] explored the potential of combined *G. intraradices, Gliocladium catenulatum, T. atroviridae and Bacillus amyloliquefaciens* to cope with *Phytophthora parasitica*, a major pathogen of *Choisya ternate* (Mexican orange). Significant reduction in the amount of disease and *P. parasitica* development resulted in the combined treatment of *G. intraradices* with *G. catenulatum* and *G. intraradices* with *T. atroviridae.*

Similarly, Singh et al. [26], demonstrated the efficiency of the bioinoculant *Pseudomonas monteilii* and *G. intraradices*) in the management of complex root disease of the tropical perennial plant *Coleus forskohlii* caused by *Fusarium, Ralstonia* and *Meloidogyne* under organic field conditions.

The 100% suppressive effect of vesicular arbuscular mycorrhizal fungi as root inoculant has a potential offer for FocR4 control, being an endophyte, it is less subjected to environmental effects. This finding is consistent with decreased incidence and severity of Fusarium crown and root rot of tomato, caused by *Fusarium oxysporum* f. sp. *radicis-lycopersici* using *T. harzianum* and *G. intraradices* and the combination of the two biocontrol agents [24]. Vesicular arbuscular mycorrhizal fungi are reported to reduce access sites of root invading pathogens and stimulate host defense and indirectly suppress plant pathogens through enhanced nutrition, increased lignifications and production of antifungal compounds chitinase and isoflavonoids [10,11,12]. *T. harzianum*, on the other hand, is known to be an antagonistic microbe [3]. *Trichoderma* spp. had provided control over diseases caused by various fungal pathogens through their various types of actions which include mycoparasitism, antibiosis, induced resistance, competition for nutrients or space, and inactivation of the pathogen’s enzymes [5]. 

## 4. Conclusion and Recommendations

The effect of the combined application of *T. harzianum* and *Glomus* spp. in reducing Fusarium wilt disease incidence was non-synergistic. Treatment with *Glomus* spp. provided the best disease control. Based on the study, we recommend the introduction of *Glomus* spp. as root inoculants as early as the seedling stage. Prior establishment in the banana roots will result in the production of networks of mycorrhizae inside the roots and in the rhizosphere. A pinch of BIOTECH product *Glomus* spp./pot applied basally during transplanting of the tissue cultured plantlets is recommended. Possibly, *T. harzianum* could be integrated after *Glomus* spp., but longer timing is needed for its establishment in the soil.

## Figures and Tables

**Table 1:** Mean plant growth (height, pseudostem diameter and root weight) as affected by the application of *Glomus* spp. and *T. harzianum* with or without FocR4 inoculation.

Treatment	Mean Plant Height Increment/Loss (cm) **	Standard Deviation	Mean Pseudostem Diameter Increment/Loss (cm) **	Standard Deviation	Root Weight (g) **	Standard Deviation
FocR4 alone	−8.3 ^d^	7.6	−1.67 ^d^	1.26	46.33	17.2
FocR4 + *T. harzianum*	3.0 ^c^	13.5	1.67 ^b^	1.04	36.67 ^c^	23.7
FocR4 + *Glomus* spp.	2.7 ^a^	10.3	1.33 ^ab^	1.04	39.83 ^bc^	4.3
FocR4 + *T. harzianum* + *Glomus* spp.	−1.3 ^c^	6.7	−0.17 ^bc^	1.53	41.17 ^a^	16.3
*T. harzianum* alone	10.7 ^b^	4.7	2.83 ^a^	1.89	31.67 ^b^	25.4
*Glomus* spp. alone	6.0 ^a^	10.8	2.17 ^a^	0.29	55.67 ^a^	13.4
*T. harzianum* and *Glomus* spp.	7.0 ^a^	20.9	2.50 ^a^	1.80	38.00 ^bc^	8.4

** Significant at 1% level of probability. Means having the same letter superscripts are not significantly different at 1% level using Tukey HSD. Mean of three replicates, height and pseudostem diameter growth increment/loss was taken as a difference between the final and initial data after six weeks.

**Table 2:** Area under the disease progress curve of Fusarium wilt incidence (units/week).

Treatment	Time (Week)				
	2	3	4	5	6
FocR4	115.5	346.5	462	581	-
FocR4 + *T. harzianum*	0	0	115.5	231	346.5
FocR4 + *Glomus* spp.	0	0	0	0	0
FocR4 + *T. harzianum* + *Glomus* spp.	0	0	0	115.5	231
